# Efficacy of Incobotulinumtoxin A on Maskne: Evaluation in a Prospective, Single‐Center, Placebo‐Controlled, Double‐Blind Study

**DOI:** 10.1111/jocd.70391

**Published:** 2025-08-23

**Authors:** Alena Roessle, Stefanie Gluecklich, Martina Kerscher, Heike Buntrock

**Affiliations:** ^1^ Cosmetic Science Institute of Biochemistry and Molecular Biology, University of Hamburg Germany

**Keywords:** botulinum toxin, COVID‐19, Incobotulinumtoxin a (INCO), mask, maskne, skin quality acne

## Abstract

**Background:**

Skin quality affects both external perception and psychological well‐being. A decline in skin quality, as seen in acne tarda or due to prolonged mask use, especially in healthcare professionals, can be reflected in emergent perceptual category (EPC) parameters. During the COVID‐19 pandemic, this led to an increase in mask‐induced acneiform eruptions, commonly referred to as “maskne.” Intradermal application of Incobotulinumtoxin A (INCO) may improve skin quality, but data in maskne patients are limited.

**Aims:**

To evaluate the efficacy of intradermal INCO on skin quality in women with maskne.

**Patients/Methods:**

In a prospective, randomized, double‐blind, placebo‐controlled trial, 36 women with maskne received intradermal INCO (20 U) or placebo (2:1) in the mid and lower face. Skin roughness (SEr), sebum level, pore size, and erythema index were measured over 112 days using biophysical tools. Patient and expert assessments were recorded via Global Impression of Change Scale (GICS).

**Results:**

Thirty‐three participants completed the study. The INCO group showed significantly greater SEr improvement than placebo on days 28, 56, 84, and 112. Sebum level decreased significantly by day 28 (−19.27 μg/cm^2^ vs. +1.27 μg/cm^2^ in placebo; *p* < 0.001). Pore size differed significantly at days 28 and 56. Erythema decreased in both groups, with greater reduction in the INCO group (−43.81 vs. −3.27; *p* = 0.041). Both patients and investigators reported improved skin quality in the treatment group.

**Conclusion:**

Intradermal injection of INCO represents a promising approach for managing mask‐associated skin conditions (“maskne”). While further investigations are necessary to fully establish its benefits, the present study offers encouraging evidence supporting its efficacy.

## 1 Introduction

Healthy skin is widely recognized as an essential component of overall well‐being and the quality of life [1, 2]. The concept of skin quality encompasses a variety of parameters that can be divided into four emergent perceptual categories (EPCs). These are: skin tone evenness, skin surface evenness, skin firmness, and skin glow [3].

These EPCs can be negatively affected by clinical symptoms such as redness, irritation, and papulopustular eruptions. Such features are observed in individuals with acne tarda. In women with adult acne, inflammatory lesions such as papules, pustules, and nodules usually appear on the lower part of the face in a U‐shaped pattern and tend to worsen during the premenstrual phase [4]. Such dermatologic alterations have also been seen in healthcare professionals who wear Filtering Face Piece (FFP) or surgical masks for extended periods [5]. During the COVID‐19 pandemic, such mask‐induced skin changes were reported with increasing prevalence, likely due to mandatory and continuous mask usage. This phenomenon has been termed “maskne”—a neologism formed by combining the words “mask” and “acne”—as it results in acneiform skin lesions within the mask‐wearing areas [6].

Several published studies have demonstrated that Botulinum NeuroToxin Type A (BoNT‐A) can reduce erythema (rosacea), seborrhea, and other signs of poor skin quality [7, 8, 9, 10, 11]. Since 2002, formulations of Incobotulinumtoxin A (INCO) containing only the purified BoNT‐A neurotoxin, free of complexing proteins, have been approved for aesthetic treatment of dynamic facial lines in the upper face regions [12, 13]. While intramuscular application reduces facial muscle activity, intradermal use has shown additional effects on skin quality, including refined texture, minimized pores, and decreased sebum levels, as demonstrated previously [9, 14, 15]. Therefore, the aim of the present study was to investigate the effects of intradermally administered INCO in the lower and midface regions on skin quality in patients affected by maskne.

## 2 Materials and Methods

The single‐center study was designed as prospective, randomized, double‐blind, and placebo‐controlled. It was conducted in accordance with the guidelines of the Declaration of Helsinki and the International Conference of Harmonization Guidelines for Good Clinical Practice. In 2021, the Independent Ethics Committee (Ethikkommission der Ärztekammer Hamburg) and the Federal Institute for Drugs and Medical Devices (Bundesinstitut für Arzneimittel und Medizinprodukte) approved the study.

A total of 36 women aged 18 to 60 years participated in a 112‐day observational period. They were included if they reported dissatisfaction with the skin quality in the lower face associated with regular mask‐wearing. Although the term “maskne” was used as a guiding concept, the condition was not regarded or diagnosed as a dermatosis, but rather as a functional or aesthetic impairment of the skin, primarily reflected in objective biophysical parameters. Participants who had received prior treatments with BoNT‐A, hyaluronic acid (HA), platelet‐rich plasma (PRP), calcium hydroxylapatite (CaHA), laser, or ultrasound in the mid or lower face within the past 12 months were excluded. In addition, pregnant or breastfeeding women were not eligible for participation. Written informed consent was obtained from all participants prior to the commencement of any study‐related procedures.

**FIGURE 1:**
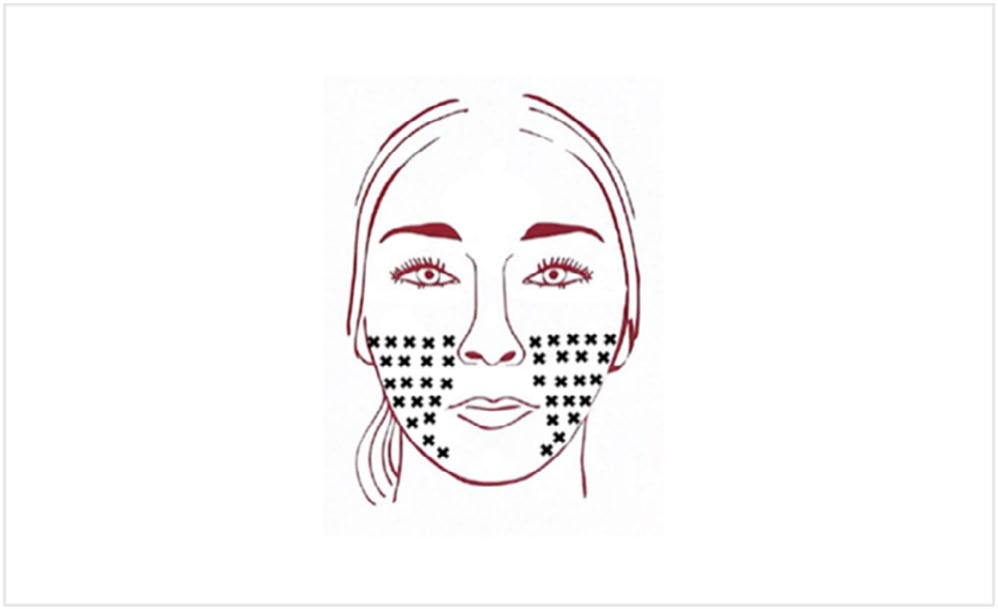
Injection scheme (0.025 mL aliquots of 0.5 U per injection point).

**FIGURE 2:**
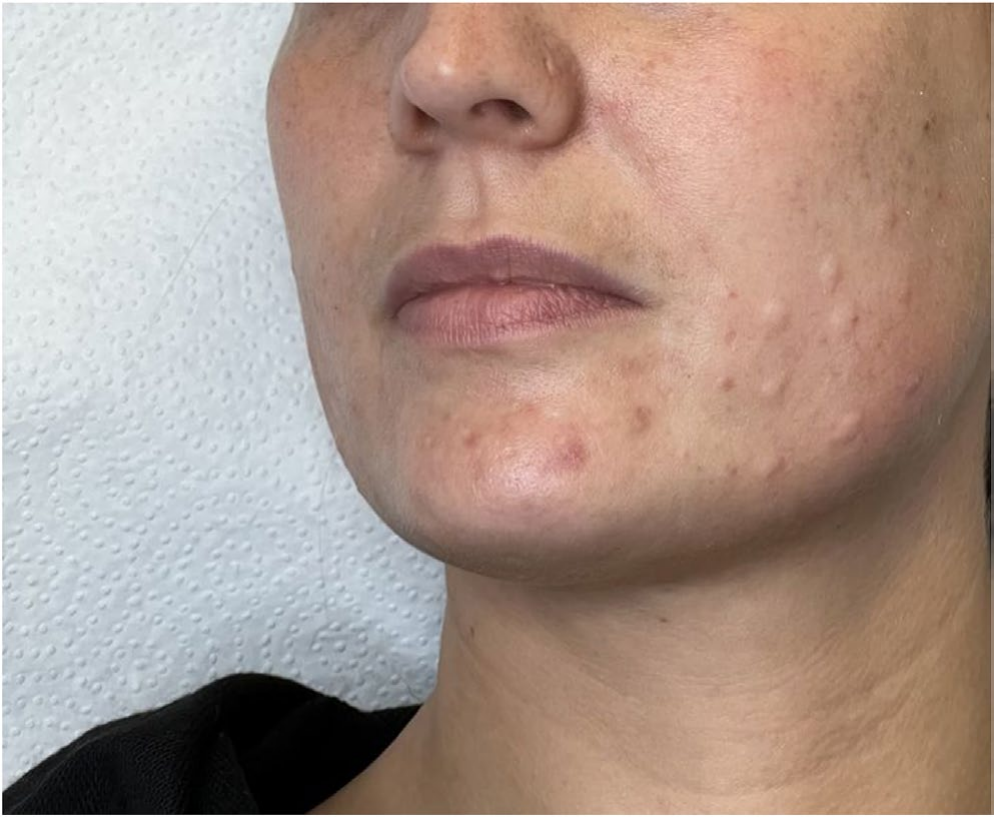
Injection technique (intradermal application).

The study included six visits in total: a screening visit conducted between −14 and 0 days before baseline, a baseline visit (day 1), and four follow‐up visits on days 28 ± 3, 56 ± 7, 84 ± 7, and 112 ± 7. At baseline, 24 subjects received an intradermal injection of a total dose of 20 U INCO (verum group), and 12 subjects received a placebo (saline solution) in the middle and lower face following a fixed injection field with 20 injection points per side (Figure 1). The INCO treatment involves the administration of 0.025 mL aliquots of 0.5 U per injection point (Figure 2). Participants were randomly assigned to one of the two treatment groups. Both the injector and the participants were blinded to group allocation. Injection volume, number of injection points, needle type, and syringe were identical in both groups to ensure procedural consistency.

Biophysical measurements were performed under standardized conditions (temperature at ~20°C, humidity at ~50%) using devices from Courage+Khazaka electronic GmbH, Cologne, Germany. Subjects were not allowed to wear face masks during the measurements to ensure standardization. Skin roughness (SEr) was assessed with Visioscan VC 20plus, sebum level with Sebumeter SM 18, pore size with VisioFace 1000 D, and erythema index with Mexameter MX 18. Furthermore, expert and patient satisfaction was assessed using the Global Impression of Change Scale (GICS) at each visit. Standardized photography (Cannon, Modell PowerShot G10) was conducted to visualize the before‐and‐after effects.

The data were analyzed using Mixed ANOVA and Welch's test. All statistical assumptions, including normality and sphericity, were tested and met. Mixed ANOVA evaluated within‐subject effects (temporal changes) and between‐subject effects (differences between the verum and placebo groups). Welch's ANOVA identified significant differences across visits, with a significance level of *p* < 0.05.

## 3 Results

In total, 33 female subjects completed the study (39.2 ± 8.9 years), with 22 receiving INCO and 11 receiving placebo. Overall, the treatment was well tolerated. Only minor adverse events included small, typical, transient needle‐induced hematomas at the injection sites in four participants, all resolving spontaneously without the need for intervention. Despite injections near the oral commissures, no lip asymmetry or ptosis was observed.

### 3.1 Skin Roughness

**FIGURE 3:**
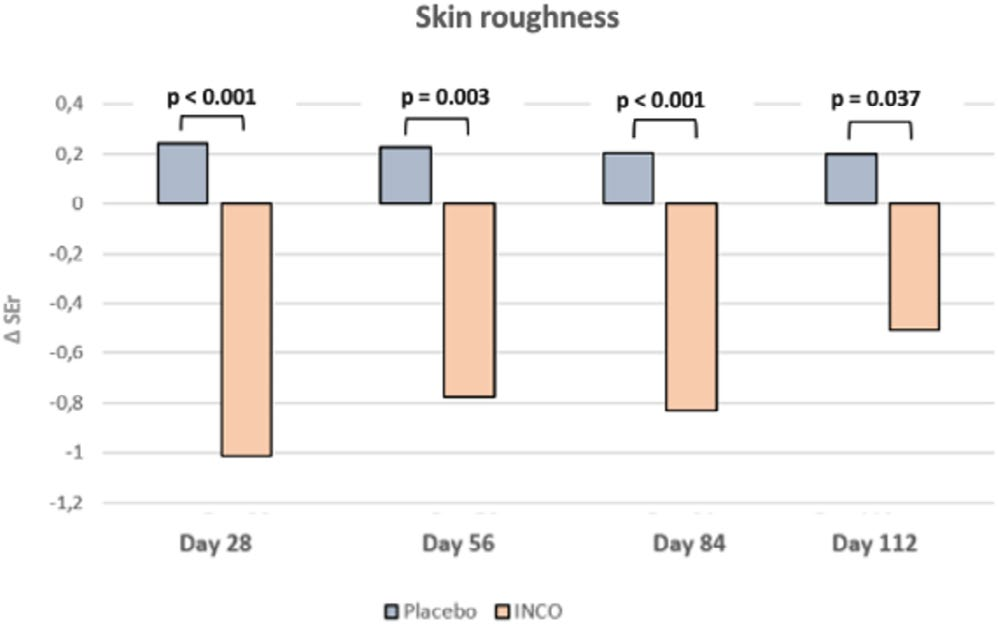
Results of SEr of the middle and lower face area.

The improvements in SEr were observed in the INCO grouped compared to placebo at all follow‐up visits. The greatest difference occurred at day 28 (INCO: −1.01 vs. placebo: +0.24; *p* < 0.001), with sustained significance at day 56 (INCO: −0.89 vs. placebo: +0.22; *p* = 0.003), day 84 (INCO: −0.83 vs. placebo: +0.20; *p* < 0.001), and day 112 (INCO: −0.61 vs. placebo: +0.20; *p* = 0.037). The SEr values in the placebo group remained stable and normally distributed throughout the study period (Figure 3, Table 1).

**TABLE 1:** Average SEr and Deltas Across Time Points.

Time points	SEr INCO	Difference to baseline (INCO)	SEr placebo	Difference to baseline (placebo)	Difference INCO and placebo *p*
Baseline	3.85	—	3.67	—	—
Day 28	2.84	−1.01	3.91	+0.24	*p* < 0.001
Day 56	2.96	−0.89	3.89	+0.22	*p* = 0,003
Day 84	3.02	−0.83	3.87	+0.20	*p* < 0.001
Day 112	3.24	−0.61	3.87	+0.20	*p* = 0.037

### 3.2 Sebum Level

**FIGURE 4:**
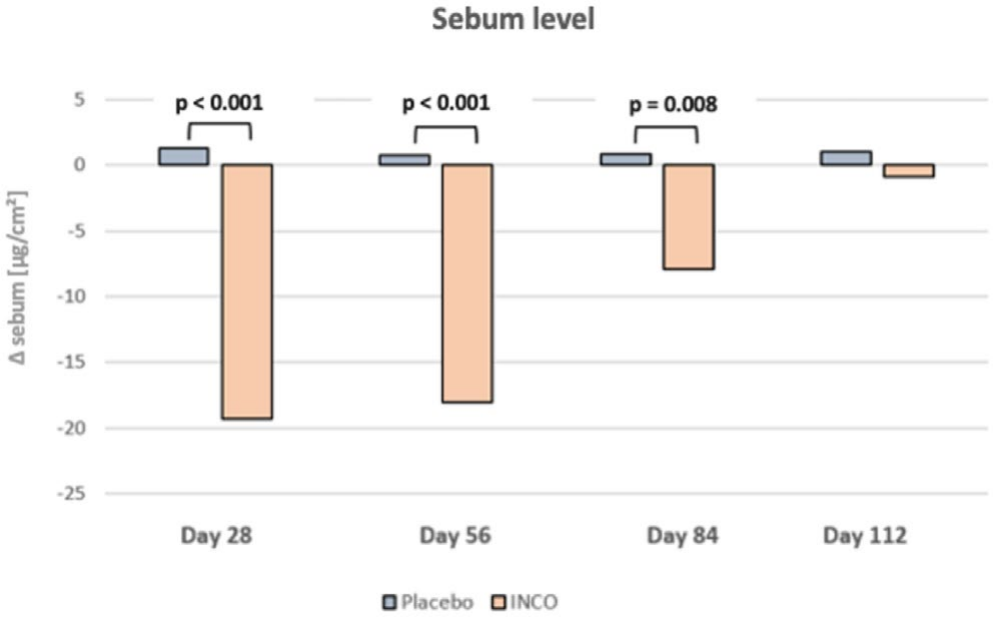
Results of the sebum level of the middle and lower face area.

Sebum levels decreased markedly in the INCO group compared to the placebo group through day 84, with no significant differences by day 112 (Figure 4). At day 28, INCO‐treated subjects showed a reduction of −19.27 μg/cm^2^ compared to +1.27 μg/cm^2^ in the placebo group (*p* < 0.001). Similar significant differences were found at day 56 (INCO: −18.09 μg/cm^2^ vs. placebo: +0.73 μg/cm^2^; *p* < 0.001) and day 84 (INCO: −7.09 μg/cm^2^ vs. placebo: +0.82 μg/cm^2^; *p* = 0.008). At day 112, the difference was no longer significant (INCO: −0.86 μg/cm^2^ vs. placebo: +1.00 μg/cm^2^; *p* = 0.41). While intradermal injection with INCO initially led to a marked reduction in sebum levels in the verum group, this effect was not observed after 112 days. The placebo group showed a slight increase in sebum levels, although the sebum values remained normally distributed throughout the duration of the study and did not exhibit significant changes (Figure 4, Table 2).

**TABLE 2:** Average Sebum Levels and Deltas Across Time Points.

Time points	Sebum level INCO	Difference to baseline (INCO)	Sebum level placebo	Difference to baseline (Placebo)	Difference INCO and placebo *p*
Baseline	115.95 μg/cm^2^	—	111.00 μg/cm^2^	—	—
Day 28	96.68 μg/cm^2^	−19.27 μg/cm^2^	112.27 μg/cm^2^	+1.27 μg/cm^2^	*p* < 0.001
Day 56	97.86 μg/cm^2^	−18.09 μg/cm^2^	111.73 μg/cm^2^	+0.73 μg/cm^2^	*p* < 0.001
Day 84	10.86 μg/cm^2^	−7.09 μg/cm^2^	111.82 μg/cm^2^	+0.82 μg/cm^2^	*p* = 0.008
Day 112	115.09 μg/cm^2^	−0.86 μg/cm^2^	112.00 μg/cm^2^	+1.00 μg/cm^2^	*p* = 0.41

### 3.3 Pore Size

**FIGURE 5:**
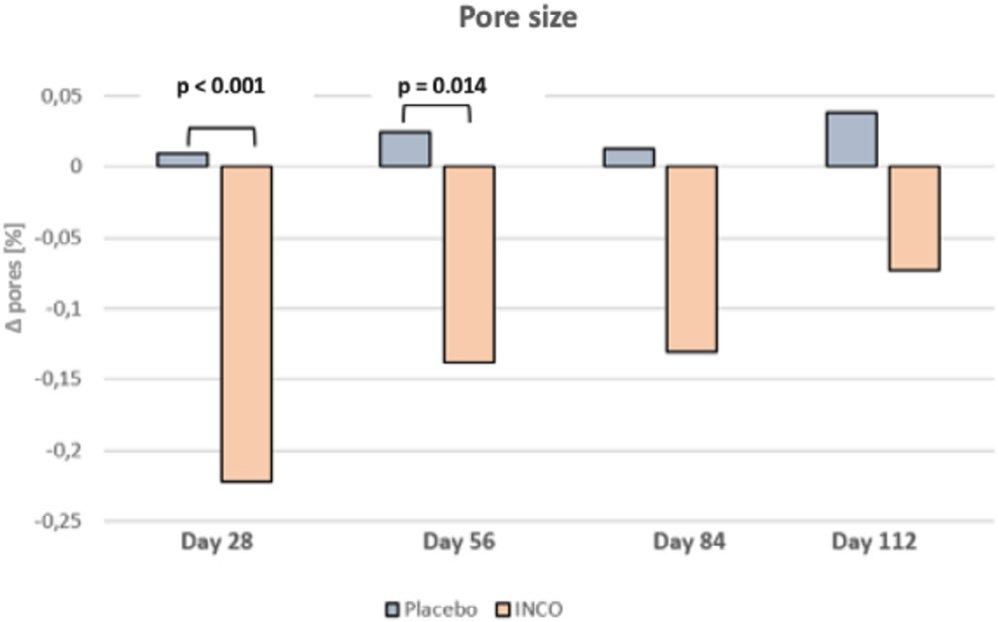
Results of the pore size of the middle and lower face area.

Pore size was significantly reduced in the INCO group at day 28 (−0.22%) and day 56 (−0.14%), while the placebo group showed minimal changes (+0.01% and + 0.03%, respectively). Differences between the groups were statistically significant at day 28 (*p* < 0.001) and day 56 (*p* = 0.014), but not at day 84 (*p* = 0.095) or day 112 (*p* = 0.091). The intradermal injection of INCO resulted in a significant reduction in pore size within the verum group after 28 and 56 days, contrasting with the placebo group where pore size remained relatively unchanged (Figure 5).

### 3.4 Erythema Index

**FIGURE 6:**
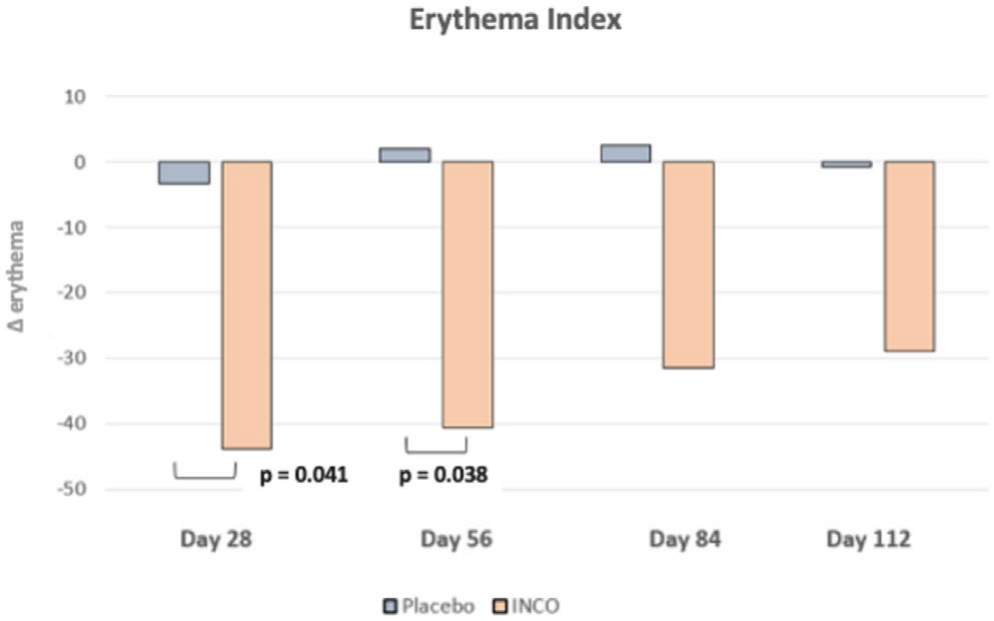
Results of the erythema index of the middle and lower face area.

The erythema index decreased significantly in the INCO group compared to the placebo group at day 28 (INCO: −43.81 vs. placebo: −3.27; *p* = 0.041) and day 56 (INCO: −40.68 vs. placebo: +2.00; *p* = 0.038). Intradermal administration of INCO led to a statistically significant decline in erythema values in the verum group at both day 28 and day 56 compared to placebo, reflecting a visible reduction in skin redness. The erythema values of the placebo group remained normally distributed between day 28 and 112 without significant changes (Figure 6).

### 3.5 Global Impression of Change Scale

Both patient and investigator reported higher satisfaction in the INCO group. GICS scores indicated significant improvement in their overall impression (+1) at day 28 (*p* < 0.001), with sustained benefit at days 56 (*p* = 0.004) and 84 (*p* = 0.004). The investigator also noted an improvement (+1) at day 28 (*p* < 0.001) and 56 (*p* = 0.001).

### 3.6 Standardized Photography

**FIGURE 7:**
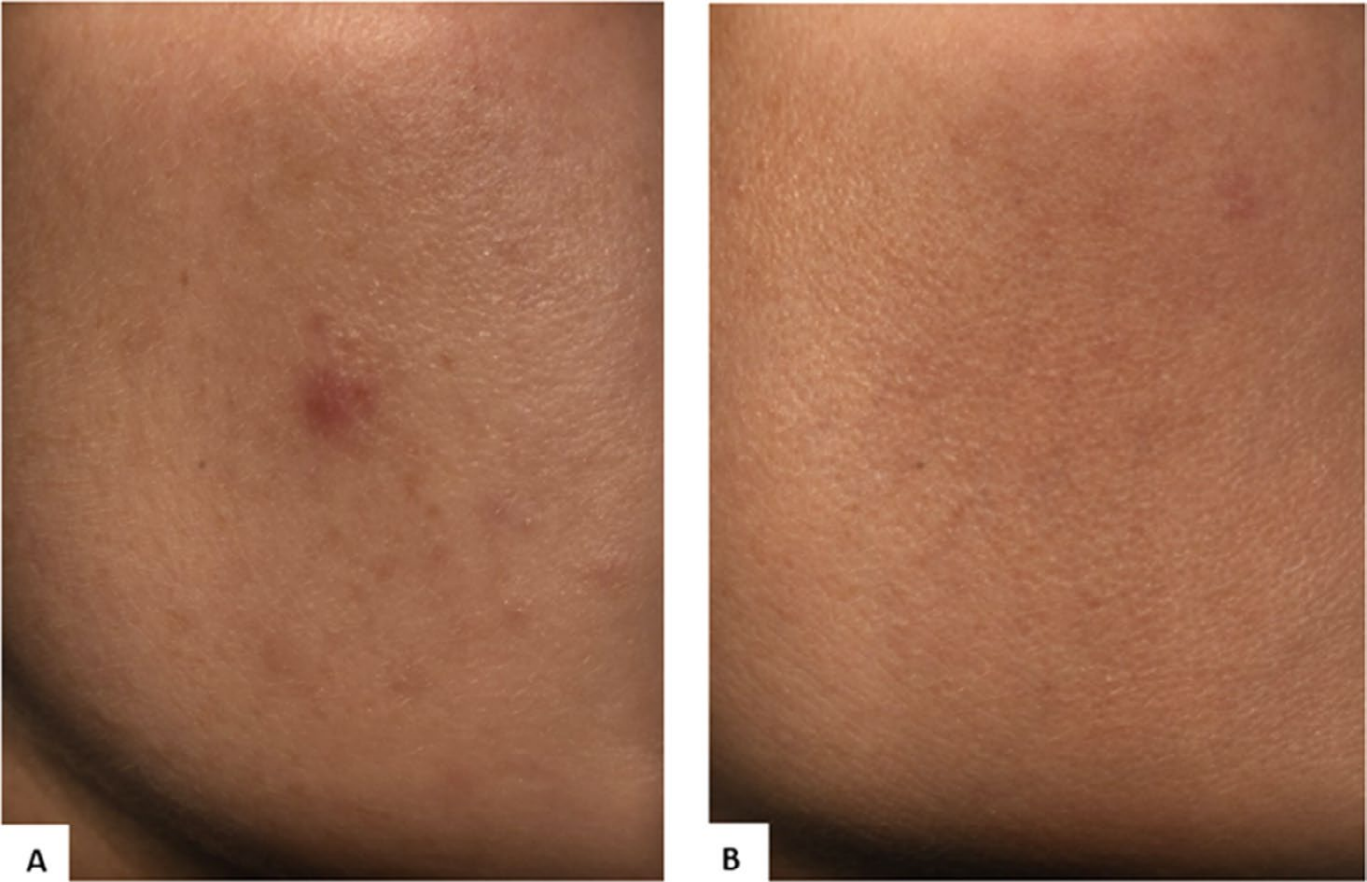
(A) Middle and lower side of the face at baseline. (B) Middle and lower side of the face 30 days after intradermal injection with INCO.

Evaluation of before‐and‐after images clearly demonstrated visible improvements in skin quality for INCO‐treated subjects. Notably, pores appeared finer, and the skin appeared smoother and more radiant (Figure 7).

## 4 Discussion

Skin quality has a significant impact on well‐being and quality of life [1, 2]. This prospective placebo‐controlled study is the first to evaluate intradermal INCO for mask‐related skin issues in Caucasian women, using objective biophysical markers mapped to established skin quality EPCs.

The intradermal application of INCO resulted in statistically significant improvements in skin surface roughness, pore size, sebum level, and erythema index, thereby contributing to an overall enhancement in skin quality. INCO significantly improved skin surface evenness (SEr), tone evenness (erythema index), and glow (sebum and pore size), aligning with three of the four EPCs [3]. The most significant effects in SEr were observed after 28 days (−1.01) compared to the placebo (+0.24), followed by a gradual increase thereafter, consistent with the known 3‐month duration of BoNT‐A efficacy [16]. These findings align with other studies that have demonstrated rejuvenation and reduced roughness in the lower facial region following intradermal BoNT‐A injection, which were tentatively attributed to the blockade of superficial fibers of the facial muscles [17, 18].

Regarding pore size and sebum production, significant differences were observed between the verum and placebo groups, persisting through days 28 and 56. Importantly, the observed reductions in sebum levels and pore size cannot be attributed solely to seasonal influences. The verum group experienced a notable decrease in pore size by day 28, while the placebo group showed a slight increase. Statistically significant differences between the groups persisted at day 56. Similarly, sebum production declined significantly in the INCO group, whereas the placebo group exhibited no meaningful change over time (+1.27 μg/cm^2^ at day 28; +0.73 μg/cm^2^ at day 56). These stable values in the placebo group support the conclusion that the observed improvements were treatment‐related and not attributed to environmental or seasonal factors. Although the study was conducted during winter, when colder temperatures might reduce sebum levels across all subjects [19], this seasonal effect was not reflected in the placebo group, further reinforcing the specificity of the INCO response.

Erythema in the middle and lower face was assessed using mexametry. By 28 days, the INCO group showed a substantial mean reduction in erythema values (−43.81), while the placebo group's reduction was minimal (−3.27). This treatment effect persisted at day 56, with the INCO group. These findings suggest that intradermal INCO not only prevents worsening of erythema but actively improves skin tone evenness relative to baseline. The result is consistent with prior studies demonstrating the efficacy of intradermal BoNT‐A in reducing erythema and flushing, particularly in patients with rosacea [20, 21]. The underlying mechanism is hypothesized to involve inhibition of mast cell degranulation and modulation of neurogenic inflammation through acetylcholine and neuropeptide pathways [22]. While promising, these findings merit further study in larger and more diverse populations to better understand the broader therapeutic potential and safety of INCO in erythema‐related conditions.

The intradermal administration of INCO has shown promising potential for improving skin quality in individuals experiencing mask‐related skin issues (“maskne”). Unlike conventional acne therapies, which primarily target inflammatory lesions, INCO was employed in this study to address biophysical skin quality parameters, such as texture, erythema, sebum production, and pore size. While prior studies demonstrating these effects have predominantly focused on Asian populations [15, 23], this is, to our knowledge, the first placebo‐controlled trial to investigate intradermal INCO in a Caucasian cohort.

INCO was explicitly selected due to its lack of complexing proteins, resulting in the lowest total protein load among currently available botulinum toxin formulations, which may support better tolerability and lower immunogenicity [24]. In the context of ongoing mask use in both professional and public settings, these findings hold significant clinical relevance. Patients experiencing skin irritation, acneiform eruptions, or diminished skin quality due to prolonged mask‐wearing may benefit from this treatment approach.

Furthermore, the improvements observed in the lower face are unlikely to be driven by superficial muscle relaxation, as the key mimetic muscles in this area—such as the buccinator or depressor anguli oris—are located deeper within the facial anatomy than the superficial muscles of the upper face, like the frontalis [25]. This supports a mechanism rooted in dermal modulation rather than neuromuscular blockade.

Overall, intradermal injection of INCO in the middle and lower face resulted in significant improvements in skin quality, including reductions in pore size and sebum production, two interrelated factors, as excess sebum contributes to the enlargement of pores [26, 27, 28]. While additional research is warranted to confirm and expand upon these findings, this study provides compelling early evidence in support of the efficacy of INCO for managing mask‐associated skin concerns. Despite limitations such as the small sample size, single‐center setting, and restriction to Caucasian female participants, this represents the first randomized, double‐blind, placebo‐controlled trial to demonstrate a positive impact of BoNT‐A on skin quality in this population. These results support the growing role of intradermal BoNT‐A as a novel, skin quality–focused intervention in aesthetic dermatology.

## Author Contributions

Alena Roessle and Stefanie Gluecklich share first authorship. Prof. Kerscher provided guidance and advisory support throughout the study process. Heike Buntrock provided support during the submission process and assisted with the revision of the manuscript.

## Ethics Statement

The authors confirm that the ethical policies of the journal, as outlined on the journal's author guidelines page, have been adhered to, and appropriate approval from the ethical review committee has been obtained. The Independent Ethics Committee (Ethikkommission der Ärztekammer Hamburg) and the Federal Institute for Drugs and Medical Devices (Bundesinstitut für Arzneimittel und Medizinprodukte) in Germany approved the study in 2021. The study was conducted in accordance with the principles of the Declaration of Helsinki and the International Conference on Harmonization Guidelines for Good Clinical Practice. Study procedures were not taken prior to the signing of the informed consent.

## Conflicts of Interest

The authors declare no conflicts of interest.

## Data Availability

The data that support the findings of this study are available from the corresponding author upon reasonable request.
